# Food security vulnerability due to trade dependencies on Russia and Ukraine

**DOI:** 10.1007/s12571-022-01306-8

**Published:** 2022-07-22

**Authors:** Petra Hellegers

**Affiliations:** Water Resources Management Group, Wageningen University & Research, Droevendaalsesteeg 3a, 6708 PB Wageningen, the Netherlands

**Keywords:** Food insecurity, Instability, Vulnerability, Resilience, Geopolitics, Global trade

## Abstract

The Russian invasion of Ukraine is disrupting global agricultural commodity markets, creating pressure on wheat supplies and stocks and consequently on food prices. The wider effects are felt around the world due to the dependencies inherent to global trade. But how to assess the vulnerability of countries food security and how to deal with it? To assess for which countries food security is at risk, dependencies along with a set of coping capacity indicators to absorb shocks need to be identified. Addressing vulnerabilities at this scale requires a global food security approach, because the food security of vulnerable countries depends on measure taken by other countries, together with a holistic approach to water, energy and food security. The Russian invasion brings to the fore the need to reassess the socio-economic value of agriculture and open trade, in terms of food security for stability in vulnerable regions.

## Introduction

A multifaceted catastrophe is unfolding with Russia’s invasion of Ukraine. Western allies have responded with successive waves of unprecedented sanctions against Russia and Belarus. Agricultural commodity markets are directly affected in various ways, such as disrupted supply chains, trade and logistics. Global cereal and oilseed markets are being hit especially hard, since Russia and Ukraine are among the leading exporters of these food commodities. Wheat is a staple food for more than 35% of the world’s population (FAO, 2022a). It is unclear whether other exporters will be able to fill the supply gap. A likely scenario is that producing countries will restrict exports in an attempt to protect domestic supply (FAO, 2022a). The fertilizers markets will be disrupted too, since Russia and Belarus are also leading exporters. This will exacerbate rises in food prices. Overall, higher prices for energy, inputs and food could have significant food security (FAO, 1996) repercussions across the world, especially in vulnerable regions (WFP, 2022).

Food insecurity is well-known to be a main source of geopolitical tensions and social unrests (Bellemare, 2015; Berazneva & Lee, 2013; Gómez, 2022; Koren et al., 2021). In the very recent past, as shown by the Arab Spring protests, food insecurity exacerbates existing frustrations and disrupts the social and political order, leading to political instability (Soffiantini, 2020). The potential for conflicts will be greater in countries with restricted trade policy space to counter the effects of a shock. Indeed, food insecurity was a main trigger for the Arab Spring protests, which began in 2011 after Russia’s grain export ban of 2010. The outcomes of the Arab Spring protests may be attributed to the food security policies, particularly for wheat, adopted by governments (Soffiantini, 2020). Appropriate approaches and measures at various scales to deal with vulnerabilities of food security to shocks are crucial.

In today’s hyper-connected global economy, with its deep trade links, many countries are more resilient than previously as local shocks can be compensated by sourcing from areas further away, although at the same time some developing countries have slipped into a vulnerable situation (Gutiérrez-Moya et al., 2021). Trade enables countries to specialize, which enables them to achieve economies of scale. It also facilitates diversification in the access to goods, services, and suppliers (WTO, 2021). However, if the shocks are of high intensity or occur over a wide region, this global sourcing cause severe vulnerability to the global trade system (Jones & Phillips, 2016). This article takes a closer look at the vulnerabilities of the global economy, countries, farmers, and consumers, to shocks in the global agricultural markets, with emphasis on the current example of Russia’s invasion of Ukraine. How to assess their resilience, or ability to absorb shocks, is illustrated using a set of coping capacity indicators. The insights gained from this analysis suggest improved approaches for addressing dependencies and the associated vulnerabilities, as well as for measures that would ensure access to scarcer food supplies for greater resilience.

## Method

To assess the vulnerability of the global economy, countries and consumers to shocks, we identified a number of indicators of dependency and coping capacity. These were then quantified, insofar as possible, using FAOSTAT data.

A first indicator is the global economy’s dependency on wheat, barley, sunflower oil and cereal exports from Russia and Ukraine and other major exporters. Global stock data on cereals can provide a measure of vulnerability to shortages (Bobenrieth et al., 2012). Although costly, the role of reserves as buffers that can dampen the effects of supply shocks is important. The stocks-to-use ratio denotes the level of carryover stock as a percentage of total demand or use, calculated as (*Beginning Stock* + *Total Production* – *Total Use*)/*Total Use*. Lower stocks-to-use ratios indicate a tighter supply and greater vulnerability.

A second indicator is countries’ dependency on wheat imports from Russia and Ukraine. We quantified the cereals import dependency ratio, which expresses how much of the domestic food supply of cereals is imported and how much comes from the country’s own production. Dependencies can also be quantified based on the concentration of export value in a limited number of countries, the potential for substitution and diversification, and the average price of exports compared to the average price of imports (UNCTAD, 2019).

The third and fourth indicators of a country’s vulnerability to shocks are its current food security and its economic and political situation. These factors play a role in, for example, a government’s capacity to expand social safety nets in the face of a shock. We quantified these based on countries’ agricultural trade balance, prevalence of food insecurity and undernourishment, and political stability.

Another interesting indicator is the share of income that consumers spend on food in a country, as this is linked to whether rising food prices could lead to social unrest. Countries differ markedly in this regard. In richer countries, consumers spend a much smaller fraction of their income on food. This ranges from less than 10%, for example, in the United States, United Kingdom and Canada, to more than 50% in countries such as Kenya and Nigeria (USDA, 2022). Even more interesting is the variance of the fraction of income spent on food within a country. In Europe, this variance is probably small; in most Sub-Saharan African countries, it probably is small too; in the USA and China, this variance can be expected to be very high, and quite high also in the West Asia and North Africa region. Both the value of the share of income spent on food and its variance are interesting indicators of threat to food security, and to civil peace. Unfortunately FAOSTAT provides no data on the share of income spent on food and its variance.

## The nature of dependencies

### Resilience and strategic dependencies

International trade dependencies were already in the spotlight before Russia’s invasion of Ukraine, due to the unprecedented shock to international trade as a result of disruption of food systems brought by the COVID-19 pandemic (OECD, 2020; Reiter & Stehrer, 2021; Savary et al., 2020). This triggered grave concerns about strategic dependencies and the resilience of global supply chains, which is their capacity to cope with vulnerabilities and absorb shocks.

Russia’s invasion of Ukraine has caused energy prices to rise, with negative consequences for the agricultural sector and wider food industry. Dependence on fertilizer imports is an area of particular significance. Russia produces 15% of the global trade in nitrogenous fertilizers, such as ammonia and urea, and 17% of the global potash fertilizers. Belarus, accounts for an additional 16% of the global potash exports (Glauber & Laborde, 2022). Supply dependence from these two countries could exceed 60% for some countries, including Ukraine (Glauber & Laborde, 2022). The price of urea is expected to increase due to sanctions and the rising price of natural gas, which is required to produce nitrogenous fertilizers.

Rising energy prices have affected more indirectly agriculture in other ways as well, such as by increasing the cost of land cultivation and irrigation (which also require energy). The manufacture of animal feeds also requires energy, alongside food processing and transport.

Russia is a major player in the global energy market, accounting for 18% of the global coal exports, 11% of the oil exports and 10% of the gas exports (FAO, 2022a). Currently, Russia supplies some 40% of the EU’s natural gas imports. Although this dependency is substantial and long-acknowledged, surprisingly it has not been considered a critical EU concern (EC, 2021). This could be because past assessments did not consider other elements of the true cost of the energetic dependency (financing a war in Ukraine by importing Russian gas and oil) It may also be that substitutions might have been considered unfeasible at the time. European importers could switch to other suppliers, such as the United States of America for exports of liquefied natural gas. However, these pose logistical challenges which add significantly to costs, suggesting that little relief is yet in sight, at least in the short term (Glauber & Laborde, 2022). Countries that rely on energy imports will face larger trade deficits and higher inflation, though oil-exporting economies will benefit from higher prices.

### Dependency on Ukraine and Russian agricultural exports

In the early 1990s, following the breakup of the former Soviet Union, the region was a net importer of cereals (Glauber & Laborde, 2022). In fact, this dependence was a driving factor behind economic reforms and the collapse of the Soviet Union, as a sharp drop in oil prices in the latter 1980s left the Soviet authorities with insufficient foreign currency to buy cereals, leading to food shortages and public discontent (Lossan, 2020).

**Fig. 1:**
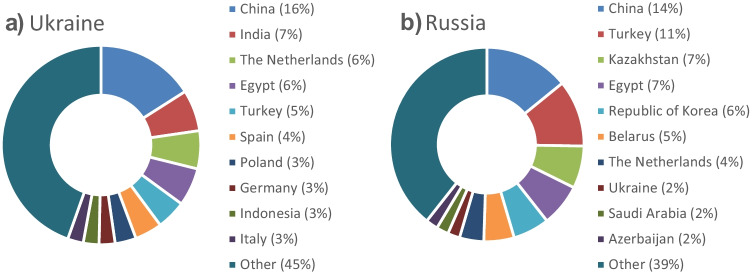
Ukraine’s major export destinations (**a**) and Russia’s major export destinations (**b**). Source: Bergevoet et al., 2022

In the past 30 years, Ukraine and Russia emerged as major global suppliers of cereals and oilseeds. The value of global agricultural exports from Ukraine, Russia and Belarus are, respectively, €19.4 billion, €24.8 billion and €5.0 billion in 2020 (Bergevoet et al., 2022). Agricultural exports from Ukraine and Russia to the EU-27 countries were valued at, respectively, €5.4 and €2.7 billion in 2020. Ukraine’s main agricultural export destinations were China, India, the Netherlands, Egypt, Turkey, Spain and Poland (see Fig. 1a). Russia’s main agricultural export destinations were China, Turkey, Kazakhstan, Egypt, the Republic of Korea, Belarus and the Netherlands (see Fig. 1b). Belarus exported three quarters of its agricultural products to Russia (Bergevoet et al., 2022).

**Fig. 2:**
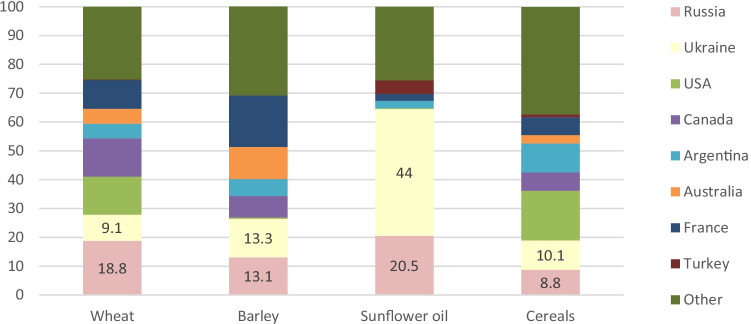
Ukraine, Russia and other main exporting countries’ shares (%) in key exports, 2020. Source: FAOSTAT, March 2022 https://www.fao.org/faostat/en/#data

Production and exports must be clearly distinguished. In 2020, the global wheat production was 760.9 million tonnes, of which Russia produced 11.3% and Ukraine 3.3%. About a quarter of the wheat produced, 198.5 million tonnes, was traded. According to FAOSTAT data, Russia and Ukraine supplied, respectively, 18.8% and 9.1% of the wheat on the global market in 2020, with the United States, Canada and France being other major wheat exporters (Fig. 2). Russia and Ukraine supplied, respectively, 13.1% and 13.3% of the barley traded globally in 2020. Ukraine supplied 44.0% of the sunflower oil traded on the global market in 2020 and Russia supplied 20.5%. This means that large sunflower oil importers will have to find other suppliers or switch to other vegetable oils, likely leading to spill-over effects on the palm, soy and rapeseed oil markets. Russia and Ukraine were among the world’s largest exporters of cereals in 2020, together with the United States, Canada, Argentina and France. Food commodity exports from Russia and Ukraine accounted for about 12% of total calories traded on the global market in 2020 (Glauber & Laborde, 2022).

**Fig. 3:**
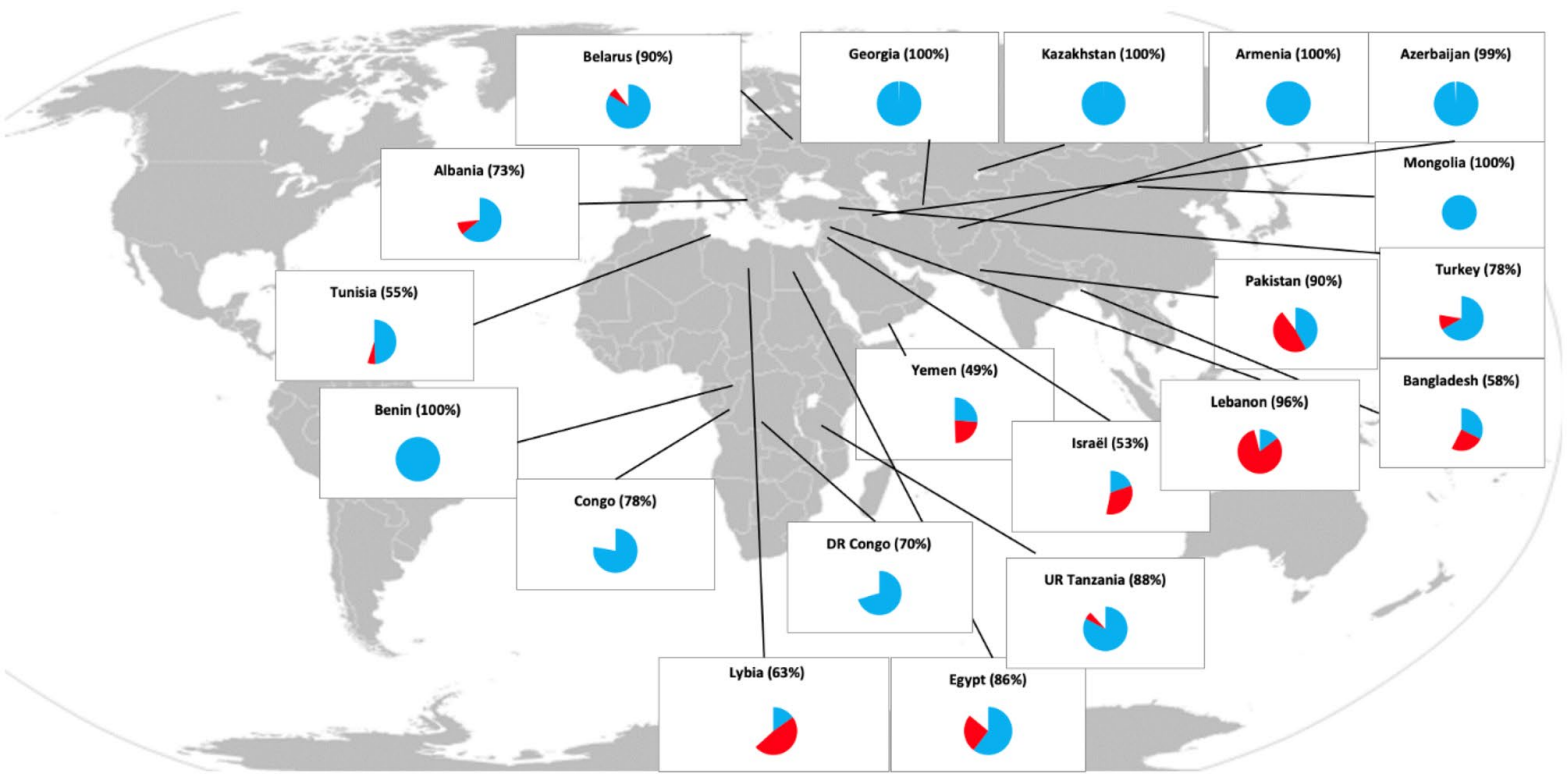
Countries share of wheat import dependencies from Russia (blue) and Ukraine (red) in 2020 (%) Source: FAOSTAT, March 2022 https://www.fao.org/faostat/en/#data

These export figures do not provide insight into the reliance of importing countries. About 50 countries import over 30% of their wheat from Ukraine and Russia (FAO, 2022a). Of these, 15 import over 70% of their wheat from these two countries (Fig. 3). Many countries in this group are highly dependent, low-income, and food-deficient countries in the Middle-East and North Africa (MENA) region.

### Coping capacity indicators of main wheat importing countries

Whether countries are resilient to shocks depends on the extent of their external dependencies and their coping capacities. Figure 3 shows countries’ import dependencies on Ukraine and Russia and Table 1 shows countries’ coping capacity indicators, which were quantified using FAOSTAT data for 2020. The trade balance of agricultural products tells us how reliant a country can be on imports. Other indicators are the prevalence of food insecurity and undernourishment, and political stability, which measures perceptions of the likelihood of political instability and/or politically motivated violence, including terrorism. This last is presented as an index with scores ranging from –4 to 2 and higher scores reflecting better situations. Although the reliability of the accuracy of those indices is questionable, they at least do give an indication of the coping capacity.

**Table 1:** Import dependency and coping capacity indicators

	Wheat^a^ import dependency ratio in 2020 (%)	Cereal import dependency ratio in 2020 (%)	Trade^b^ balance of agricultural products in 2020 (million US$)	Prevalence of moderate or severe food insecurity in the total population 2018–2020 average (%)	Prevalence of under-nourishment 2018–2020 average (%)	Political stability and absence of violence terrorism (index)
Albania	50.7	35.2	-552	33.8	3.9	0.12
Armenia	72.6	66.8	-126	12.7	3.4	-0.51
Azerbaijan	42.9	32.7	-961	8.9	< 2.5	-0.68
Bangladesh	85.4	12.1	-10,789	31.9	9.7	-0.92
Belarus	-0.1	3.0	1560		< 2.5	0.29
Benin	100.0	33.1	-120		7.6	-0.35
Congo	100.0	90.8	-522	88.3	37.7	-0.89
D.R. Congo	97.6	15.9	-515	69.2	41.7	-1.81
Egypt	50.1	42.5	-8038	27.8	5.4	-1.07
Georgia	82.7	61.6	-249	39.7	8.7	-0.45
Israel	93.2	94.2	-4450	13.7	< 2.5	-0.78
Kazakhstan	-47.1	-66.4	-628	2.3	< 2.5	-0.08
Lebanon	81.8	88.7	-1483		9.3	-1.64
Libya	89.7	93.1	-3422	37.4		-2.57
Mongolia	30.4	39.3	-397	26.2	4.3	0.64
Pakistan	8.9	-3.8	-4486		12.9	-2.25
Tunisia	65.7	71.8	-875	25.1	3.0	-0.83
Turkey	31.7	18.4	3049		< 2.5	-1.34
U. R. of Tanzania	89.8	2.3	856	56.4	25.1	-0.36
Yemen	96.8	91.6	-4135		45.4	-2.77

Source: FAOSTAT, March 2022 https://www.fao.org/faostat/en/#data

^a^Calculated as (wheat imports – wheat exports)/(wheat production + wheat imports – wheat exports)*100

^**b**^Calculated as (total export value of agricultural products – total import value of agricultural products)

Net food importing countries are more vulnerable to the effects of the Russian invasion if they have a high cereal import dependency ratio and a high reliance on cereal imports from Ukraine and Russia. Figure 3 shows that Pakistan depends on Ukraine and Russia for about 90% of its wheat imports, but Table 1 shows that this accounts for less than 9% only of its actual, available domestic wheat-based food supply. By contrast, Egypt depends on both counties for 86% of its wheat imports (Fig. 3), and this represents over 50% of its available domestic wheat-based food supply (Table 1). Similarly, Lebanon depends on Russia and Ukraine for 96% of its imported wheat (Fig. 3), and has a wheat import dependency ratio of 81.8% (Table 1).

Plotting the cereal import dependency ratio and the share of exports from Ukraine and Russia in total cereal imports for various countries (Weil & Zachmann, 2022) shows that the MENA countries which relied almost strongly on imports from Russia and Ukraine to sustain their cereal-based food consumption are especially vulnerable. Countries most at risk are those, such as Egypt, Yemen, Tunisia, and Lebanon, which not only have a high dependency, but also have limited coping capacity. Their agricultural trade balance deficits are substantial, they face moderate or severe food insecurity and undernourishment, and they suffer from political instability. Food insecurity and undernourishment is also a critical, poorly documented, and widely variable threat for African countries, such as Congo and Tanzania (Table 1).

## Results

Global production and trade figures suggest that the world is unlikely to face an immediate food shortage. Russian and Ukraine exports combined account for some 19% of the cereals traded on the global market, and the global cereal stocks-to-use ratio for 2020–2021 is 28.4% (Bobenrieth et al., 2012; FAO, 2022a). There are still wheat stocks in warehouses around the world and crops awaiting harvest. Besides, roughly 14% of global food production is typically lost between harvest and retail, while 17% annually is wasted in households, food service and retail (UN, 2021). It also worth noting that adopting a plant-based diet would reduce global cereal demand considerably (Springmann et al., 2018). Nevertheless, global food prices will inexorably rise with the increased demand for substitutes and the higher cost of food production, processing and transportation, which result from rising energy prices.

Food availability is currently not at stake in the EU (EC, 2022), because the EU is a net food exporter, and imports from Ukraine account for just 5% of the EU’s total agricultural imports. Nonetheless, the EU has substantial dependencies in certain other commodities, such as sunflower and rapeseed oil, of which the EU imports, respectively, 88% and 41%. The EU will also experience indirect effects. For example, Ukraine is a key exporter of maize, which is an important ingredient in the animal feed required for the intensive livestock farming in Europe. Global maize trade will decline if the halting of exports from Ukraine is not replaced by other exporters. This illustrates the complexity of the vulnerabilities of the EU industry.

There are major concerns in Ukraine regarding crop establishment, irrigation, input supply and access, harvests, logistics and destruction of infrastructure. These are critical to Ukraine’s food security itself (FAO, 2022b). Spring barley must be sown in March, maize in April and winter wheat in September; and cereal crops have in general to be harvested typically by June. However, Ukraine’s farmers encounter difficulties in accessing fields and sourcing labour, since millions of people fled the country or joined the fight.

Another immediate concern is the food security for the net food importing, low-income countries that are highly dependent on Ukraine and Russia for cereal supplies, such as a number of MENA countries. These countries, where many inhabitants are food insecure and malnourished, partly due to pandemic disruptions, have large and growing populations to feed. These countries also often have trade balance deficits. These countries are also characterised by income use patterns strongly assigned to daily food purchase. The Ukraine conflict could amplify these existing frailties, with very serious consequences. Among those of greatest concern are Yemen, which is in a war, Lebanon, which is in the midst of a severe economic slump, and also Egypt and Tunisia, which have faced an upsurge in food prices for months. Another is Turkey, which is experiencing a severe economic crisis.

## Discussion on approaches and measures to deal with vulnerabilities

International organizations have responded with a number of actions and policy recommendations to eschew or absorb the shock. The International Food Policy Research Institute (Glauber & Laborde, 2022) recommends that both the sanctions on Russia and export restrictions to protect domestic consumers be designed to protect global food security, and that consequences for third parties be assessed. The European Commission (EC, 2022) will help Ukraine to continue growing cereals and oilseeds by ensuring that inputs reach farmers where possible. It is also committed to take all necessary measures to ensure that the EU contributes to global food security, particularly in Ukraine, North Africa and the Middle East, as well as in Asia and sub-Saharan Africa. It will also support consumers and farmers in the EU in light of rising food prices and input costs. FAO (2022a) recommends (a) keeping the global food and fertilizer trade open, (b) finding new and more diverse food suppliers while relying on existing food stocks and diversifying domestic production, (c) expanding social safety nets to protect vulnerable people, (d) avoiding ad hoc, immediate and unilateral policy reactions and (e) strengthening market transparency and dialogue. Though export restrictions could help resolve domestic food security challenges, they will also inexorably drive global market prices up. Governments are therefore called upon to consider how their own measures to safeguard domestic food supply by restricting or banning exports might affect international markets.

A global food security approach is critically required when designing sanctions and domestic export restrictions in order to avoid further rises in global market prices and regional food insecurity. If such effects cannot be avoided, then mitigation packages are required for the affected third-party countries. These packages should include measures for managing food price risks, such as keeping cereal reserves that can dampen the effects of supply shocks, because this is the single-most important short-term driver of conflicts and unrests. In wealthy countries, governments can bear the burden of subsidizing energy or food prices or lowering taxes on energy or food.

A holistic approach to water, energy and food security is also critical. It requires developing integrated methods that consider all stakeholders, policy makers and the complexities of water-energy-food systems to contribute to reducing trade-offs and increasing synergies for long-term and sustainable decisions (Zarei, 2020). Sanctions on Russian gas will for instance drive natural gas prices up, leading to wider supply-chain disruptions. Reliance on Russian energy calls for greater energy sovereignty and diversification, for example, by biofuel production. However, redirecting food crops such as maize to non-food uses can have perverse consequences, including generating additional tensions in commodity markets and create an extra strain on food security.

Consumers should stay calm and avoid hoarding, as otherwise prices will spike (Timmer, 2022). Some level of trust in world cereal markets and governments to deliver the needed supplies in time is necessary. Such trust depends on the cooperation among stakeholders. In this regard, the rice crisis of 2008 offers a valuable parallel. The rice crisis was caused by panicked importers and exporters, along with the hoarding by small-scale players (e.g., mills) in the rice supply chain, causing unprecedented price spikes. And yet, the global rice market stabilized and rice prices fell in a matter of weeks after Japan announced that two million tonnes of US rice would be available for re-export from Japanese stocks (Timmer, 2022). Full, and open, accounting of current food stocks by the main exporters could prevent these 2008 tragic events to repeat themselves. A pledge from large cereal exporters to allocate supplies to countries most in need could reduce importer’ fears, build trust, and so stabilize the world market ahead of an impending crisis.

The discrepancy between the low financial value of food production – which is often taken for granted– and the high socio-economic value of food security – for social stability – highlights the importance of investing in agriculture while safeguarding the open and trade-based economy (Hellegers & van Halsema, 2019). Policies aimed at increasing resilience by unwinding trade integration – for example by transferring production of strategic importance that were moved to another country back to the country from which it was originally relocated and promoting self-sufficiency – can have the exact opposite and perverse effect: effectively reducing resilience (WTO, 2021). The supplying countries lose from it, so it is in their interest to work towards resilient supply chains. In the long run, supply chain resilience must be improved by (1) providing better, open, information on potential concentrations and bottlenecks along the global supply chains, (2) imposing stress tests for supply chains, and (3) by engaging in strategic stockpiling of certain commodities.

## Conclusions

As the world has become more vulnerable to shocks, and because such shocks are bound to occur more frequently, for a number of reasons, but at least because of climate change and major climate events, there is a need to provide insight into how to assess who is at risk and how to deal with vulnerabilities to become more resilient.

The consequences of the war in Ukraine go beyond direct dependency on just wheat and sunflower oil imports from Russia and Ukraine at the European scale: a multiple-commodity crisis is about to unfold with major food, social, economic and political consequences. Other global agricultural commodity markets than just wheat and sunflower oil will be affected, due to demand for substitutes and higher cost of agricultural production, processing and transportation as a result of rising energy prices. There will also be spill-over effects of instability and social unrest in vulnerable regions due to higher food prices, which can lead to escalating conflicts and refugee flows.

To assess who is most at risk, such dependencies and spill-over effects together with a set of coping capacity indicators to absorb shocks need to be considered. The analysis shows that Russia’s invasion of Ukraine will affect food security in countries with limited coping capacity, with impacts that are anticipated to be especially concentrated in the MENA region and Sub-Saharan Africa (especially the horn of Africa), which can lead to regional instability. It is, however, also in the interest of other countries to avoid this and work towards resilience, at the very least because it may lead in the short term to refugee flows and further trade disruption. In the medium-term (a year or two at most) economies will also be gravely affected in the Global South, possibly with major consequences on the global economy.

It therefore is in the common interest to develop approaches and measures to absorb such shocks, with a holistic perspective on global food security and energy and water. Thus, governments should consider how sanctions against Russia and measures taken to secure national food supply affect international markets and vulnerable regions. This is because, as this review shows, the food security of vulnerable countries of the Global South (but other countries as well) largely depends on measures taken by others in an all-connected-world.

The current crisis brings to the fore the need to reassess the socio-economic value of agriculture and open trade, in terms of food security for stability in vulnerable regions and the water-energy-food nexus. These aspects are of critical importance in identifying strategic dependencies and designing sanctions.
